# Three-dimensional changes in mandibular jaw cysts following decompression surgery: a retrospective cohort study

**DOI:** 10.1186/s12903-026-07729-5

**Published:** 2026-01-27

**Authors:** Clemens Raabe, Valerie Schmidt, Martina Schriber, Vivianne Chappuis, Valerie G. A. Suter

**Affiliations:** 1 Department of Oral Surgery and Stomatology, School of Dental Medicine, University of Bern, Freiburgstrasse 7, Bern, CH-3010 Switzerland; 2 Department of Oral Surgery and Implantology, Goethe University, Carolinum, Frankfurt am Main, Germany; 3 Clinic of Cranio-Maxillofacial and Oral Surgery, Center of Dental Medicine, University of Zurich, Zurich, Switzerland; 4 Department of Oral Health & Medicine, University Center for Dental Medicine Basel UZB, University of Basel, Basel, Switzerland

**Keywords:** Jaw cyst, Decompression, Surgical, Cone-Beam Computed Tomography, Bone Remodeling, Radicular Cyst, Dentigerous cyst, Odontogenic Keratocyst

## Abstract

**Background:**

This retrospective cohort study primarily assessed three-dimensional changes in mandibular jaw cysts over time following decompression surgery. Secondary aims included evaluating factors potentially affecting the monthly reduction rates and assessing intra- and interexaminer reliability in the measurements.

**Methods:**

Patients who underwent decompression therapy for mandibular cysts and histopathological diagnosis were included. Cone-beam computed tomography (CBCT) scans obtained before decompression surgery (baseline CBCT) and prior to enucleation surgery (follow-up CBCT) were analyzed using a specialized 3D software to measure cyst dimensions based on preview and final lesion outlines by two examiners. Patient demographics, cyst types, localization, and cortical bone perforation were recorded and evaluated for their influence on dimensional change rates.

**Results:**

Forty patients (15 females and 25 males) with a median age of 47 years (range: 11 to 76 years) and treated with decompression of radicular (*n* = 10), dentigerous (*n* = 14), botryoid (*n* = 1), or odontogenic keratocysts (*n* = 15) were analyzed. The lesions were predominantly located in the posterior mandible (*n* = 32) and exhibited single (*n* = 14), multiple (*n* = 17), or no cortical bone perforations (*n* = 9). Mean lesion volumes decreased from baseline (3764 mm³) to follow-up (1413 mm³, *p* < 0.0001), corresponding to an average monthly volumetric reduction rate of -5.2%, without significant differences between the cyst types. Female and younger patients showed higher reduction rates (*p* ≤ 0.04), whilst localization and cortical bone perforation did not significantly influence the dimensional changes. Both inter- and intraexaminer reliability of measurements were high (intraclass correlation coefficients > 95%).

**Conclusions:**

Decompression surgery is an effective first-stage treatment to reduce the dimensions of large mandibular odontogenic cysts. Within a range of limited clinical relevance, patient-related factors such as sex and age may influence dimensional changes, whereas lesion-specific characteristics did not. Cyst measurements on CBCT were consistent across observers, indicating good reproducibility.

## Background

Jaw cysts are common pathological bone cavities based on developmental or inflammatory origin and were classified into odontogenic and non-odontogenic cysts prior to 2022 [1]. Across the entities, radicular cysts (RC) are the most frequent, followed by dentigerous cysts (DC) and odontogenic keratocysts (OK) [2–5]. DC and OK have their predilection site in the posterior region of the mandible [6, 7]. Jaw cysts often grow to significant sizes before detection and/or presentation of clinical symptoms including swelling, asymmetry, and occasionally pain or discomfort [4, 8].

Typically, jaw cysts are incidental findings in conventional two-dimensional (2D) imaging such as on panoramic or periapical radiographs. These lesions appear as osteolytic areas, with or without displacement of surrounding structures and/or teeth [4]. However, with the advent of cone-beam computed tomography (CBCT), three-dimensional (3D) imaging has demonstrated superior diagnostic value, particularly for larger cysts or those in proximity to critical anatomical structures [9–11]. CBCT in combination with specialized software packages enables a more detailed and advanced evaluation of cyst morphology, location, and dimensions, providing essential information for comprehensive treatment planning [10, 12, 13]. In selected cases where 2D imaging is insufficient for evaluation of healing processes during follow-ups, CBCT is also indicated in monitoring bone apposition within the lesion [9, 11, 13, 14].

Therapeutic approaches for jaw cysts include marsupialization, decompression, enucleation, resection, or a combination of these techniques. The selection of the therapeutic approach is determined by factors such as cyst size, location, involvement of critical anatomical structures, and patient’s age [8, 15, 16]. While direct enucleation is typically the preferred treatment, it may not be advisable for large cysts in the mandible due to risks such as paresthesia, jaw fracture, tooth devitalization or tooth loss [17, 18]. In such cases, decompression is often the first-stage treatment. This procedure involves creating an opening (corticoectomy) into the cystic cavity, performing simultaneously an incisional biopsy of the cystic membrane, and inserting a drainage device (tube, stent, or gauze) to maintain the opening [18, 19]. Decompression reduces intracystic pressure, promotes bone apposition, and leads to a reduction in cyst volume, thereby facilitating safer second-stage enucleation [8, 17, 20, 21]. The rate of cyst shrinkage following decompression determines the interval between first and second stage surgery, influencing the overall treatment duration. Factors such as initial lesion dimensions and patient’s age have been shown to affect reduction rates, with younger patients and larger cysts exhibiting more significant reductions, although most studies report data based on 2D imaging [10, 14, 17, 19, 22–25]. Additionally, cyst type may influence a lesion’s response to decompression therapy, as these lesions differ in histopathological features, including growth patterns and wall structure. Only limited information exists on whether cyst type influences 3D volumetric reduction rates in jaw cysts undergoing decompression therapy, which could support clinicians in decision-making and optimizing treatment planning.

Therefore, the primary aim of this study was to investigate the dimensional changes of mandibular jaw cysts over time following decompression surgery regarding volumes, surfaces and diameters, using CBCT scans analyzed with a specialized software. Secondary aims included assessing the influence of factors such as patient’s age and gender, localization, baseline dimensions, and cortical bone perforation on the dimensional reduction rates. Additionally, inter- and intraexaminer agreement and the time required for the measurements including software analysis was evaluated.

## Methods

This retrospective study was conducted in the Department of Oral Surgery and Stomatology at the University of Bern, Switzerland, between November 2021 and July 2023. It utilized anonymized health-related data and evaluated CBCT datasets of patients, with general consent provided for further use of medical data. The study was approved by the local institutional review board (KEK-BE: 2019 − 01595, Cantonal Ethics Commission [Kantonale Ethikkommission], Bern, Switzerland), adhered to the Declaration of Helsinki (2013), and complied with STROBE (Strengthening the Reporting of Observational Studies in Epidemiology) guidelines.

### Patient selection

The records of patients who underwent decompression therapy for mandibular cysts between January 2012 and December 2018 were reviewed electronically to assess the following inclusion criteria: (1) baseline CBCT prior to the first-stage surgery (decompression), (2) a follow-up CBCT prior to the second-stage surgery (enucleation), and (3) confirmation of diagnosis through histopathological examination following both surgeries. Exclusion criteria included incomplete visibility of the cystic lesion within the field of view (FOV) or the presence of other pathologies.

Information collected included patient demographics (age and gender), histopathological cyst type (radicular, RC; dentigerous, DC; odontogenic keratocyst, OKC; botryoid, BC), and the time interval between the baseline and follow-up CBCT.

### Clinical procedure

#### Baseline CBCT and First-Stage surgery

During the pre-surgical examination, findings on conventional radiographs indicated the need for three-dimensional imaging, which was performed using CBCT scans (3.0–7.0 mA, 80–90 kV, 10.5–17 s, 3D Accuitomo 170, J. Morita, Kyoto, Japan) with a field of view (FOV) of 4 × 4 cm to 17 × 12 cm.

The decompression surgery was performed under local anesthesia, with additional sedation or general anesthesia as needed. A mucoperiosteal flap was raised, and, if necessary, an osteotomy was conducted to fenestrate the cortical bone for access to the lesion. An incisional biopsy of the cystic membrane was obtained, stored in a formalin 4% solution and sent for histopathological examination. Wound closure was achieved with single-interrupted sutures, and decompression was facilitated by inserting iodoform gauze to drain the lesion cavity through the wound margins. Postoperative analgesics were prescribed. Sutures were removed after 7 to 14 days.

#### Postsurgical Course, Follow-Up CBCT, and Second-Stage surgery

During the postoperative period, the lesion site was assessed monthly. The area was irrigated with sterile saline, and the iodoform gauze was replaced. Periapical radiographs were taken at the clinician’s discretion to monitor bone formation within the lesion. When indicated, a follow-up CBCT was performed to evaluate the lesion’s size and the demarcation of adjacent anatomical structures.

The enucleation surgery was conducted under similar conditions as the first procedure and concluded with the complete removal of the cystic tissue, followed by primary wound closure without drainage. The enucleated tissue was again sent for histopathological analysis.

### Digital assessments

All radiographic analyses were performed using a specialized software package (Volume Designer Modul, SMOP 2.17.1, Swissmeda, Baar, Switzerland) on a 19-inch Eizo Flexscan monitor (Eizo Nanao, Wädenswil, Switzerland) by two independent examiners (V.S and V.G.A.S). Initially, both examiners conducted and discussed measurements on five randomly selected CBCT scans for calibration purposes; this data was not collected.

**Fig. 1:**
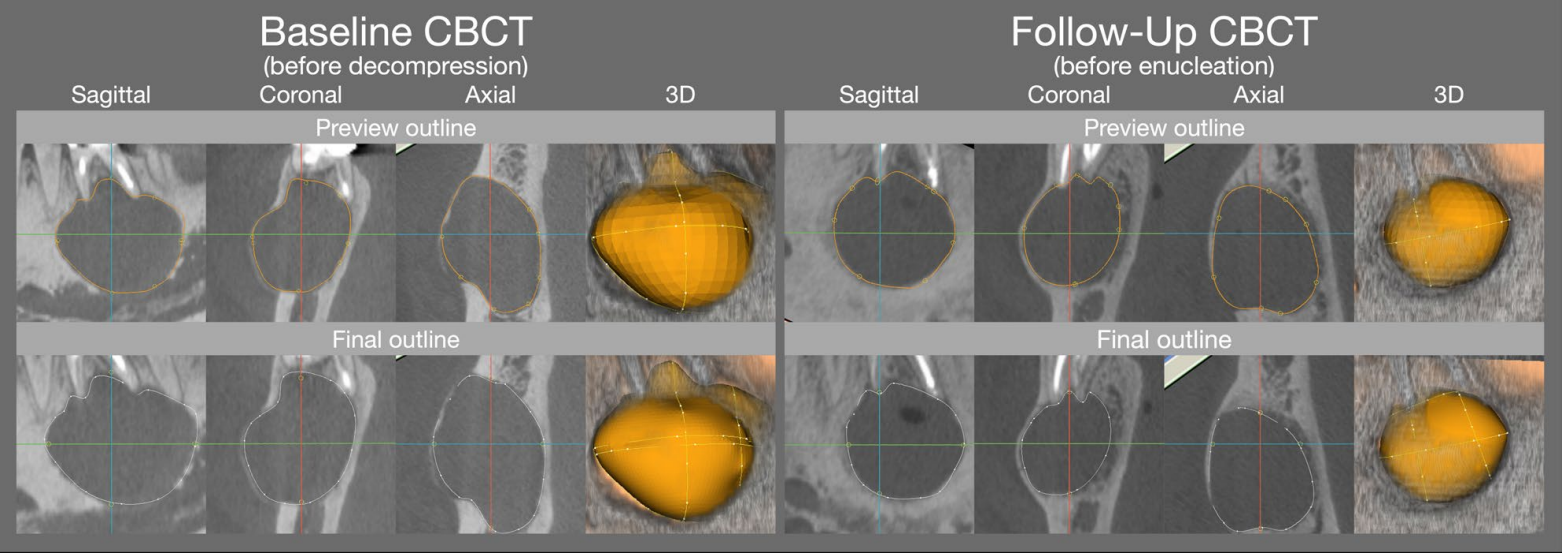
Representative case demonstrating the preview and final outlining on the baseline and follow-up CBCT, facilitating the calculation of the volumetric, surface area and maximum diameter dimensions

Subsequently, all CBCTs were analyzed regarding the following specifications (V.S): (1) localization of the lesion (anterior: canine to canine, or posterior: premolar/molar region), (2) morphology of the lesion (unilocular or multilocular), and (3) perforation of the cortical plate and its localization (none, buccal, lingual, and/or crestal, with multiple perforation sites possible). To assess the dimensions of the lesion, the contours were outlined in the coronal, sagittal, and axial planes, allowing for a preview of the reconstructed volume. This reconstructed volume was reviewed by the observer and adjusted, if necessary, to precisely determine the final volume reconstruction. Both the preview and final volume reconstruction was then used by the software’s algorithms to automatically calculate the lesion’s volume, surface area, and maximum diameter (Fig. 1). The analysis time for each case was measured from (1) the start of the initial outlining until the preview of the reconstructed volume and (2) from the first adjustment until the final volume reconstruction.

All linear and volumetric measurements were repeated after one month to assess intra-rater reliability. Additionally, a second examiner (V.G.A.S) performed the measurements once.

### Sample size calculation

An a priori sample size calculation was performed based on dimensional changes during decompression reported in a previous study [19]. To detect a statistically significant difference in one-way ANOVA with a power of 80% and a significance level of 0.05, a minimum of nine patients per cyst type was required. This resulted in a total calculated minimum sample size of 36 patients. To further maximize statistical power and reliability, all patients fulfilling the inclusion criteria were enrolled in the investigation.

### Statistical analysis

All analyses were conducted using the statistical software R (version 4.0.2, R Foundation for Statistical Computing, Vienna, Austria). The collected data were summarized descriptively using statistics such as mean, SD, minimum, Q1, median, Q3, and maximum, presented in boxplots and Table 95% confidence intervals (95% CIs) for the means were calculated using standard t-interval methods. Reproducibility of the measurements was assessed by calculating intraclass correlation coefficients (ICCs) using a two-way mixed model assessing single outcome agreement. The ICCs were classified according to Cicchetti’s guidelines [26]. The lesions dimensional change over time was determined from the dimensional differences between baseline and follow-up CBCT, pooled over both examiners, and divided by each patient’s treatment duration. As outliers with possible high leverage were detected, robust univariate linear regression models were used to analyze the effect of patient age and gender, cyst type, localization, baseline dimensions of the lesion and cortical plate perforation on the dimensional changes over time. P-values of regression models as well as confidence intervals for modelled effects were obtained by analysis of variance (ANOVA) and t-tests. Goodness of fit of regression models was checked with the help of the Breusch-Pagan test (homoscedasticity) and by visually assessing residuals and leverages of robust regression. Processing time between the two raters was assessed with the help of a paired t-test and normality of data was confirmed using the Shapiro-Wilk test. P-values less than 0.05 were considered statistically significant.

## Results

The study sample included 40 patients (15 females and 25 males) with a median age of 47 years (range: 11 to 76 years). The cystic lesions were predominantly located in the posterior mandible (80%) and had a unilocular presentation (87.5%). The lesions were classified as OKC (*n* = 15), DC (*n* = 14), RC (*n* = 10), and BC (*n* = 1), with histopathological diagnoses consistent across samples obtained at first- and second-stage surgery. Radiographically, cortical bone perforations were observed crestally (*n* = 19), lingually (*n* = 17), and buccally (*n* = 13). Single perforations were noted in 14 cases, while multiple perforations were present in 17 cases. Nine of the 40 cysts exhibited no cortical bone perforation (Table 1).

**Table 1:** Absolute and relative distribution of the sample by cyst type, localization, morphology, and cortical bone perforations. (1) cortical bone perforations were identified as single in 14 cases and multiple in 17 cases. OKC: odontogenic keratocyst, DC: dentigerous cyst, RC: radicular cyst, BC: botryoid cyst

Type of cyst (*n* = 40)		*n*	%
OKC		15	37.5
DC		14	35.0
RC		10	25.0
BC		1	2.5
**Localization** (*n*** = 40)**			
Anterior, total		8	20.0
RC		5	12.5
OKC		3	7.5
Posterior, total		32	80.0
DC		14	35.0
OKC		12	30.0
RC		5	12.5
BC		1	2.5
**Morphology** (*n*** = 40)**			
unilocular		35	87.5
multilocular		5	12.5
**Cortical perforation** (*n*** = 40)**			
Yes^1^		31	77.5
Crestal^1^		19	47.5
Buccal^1^		13	32.5
Lingual^1^		17	42.5
No		9	22.5

### Radiographic assessment

The dimensions of the lesions between baseline and follow-up decreased from 3764 mm³ to 1413 mm³ for volume, from 1392 mm² to 665 mm² for surface area, and from 30 mm to 20 mm for maximum diameter, respectively (*p* < 0.0001). Consistently, the outline for the software’s preview was consistently smaller compared to the adjusted final outline, with deviations of 2.7% to 8.5% for volume, 3.6% to 5.8% for surface area, and 0% to 2.9% for maximum diameter, respectively. The correlation coefficients for the volume, surface area, and maximum diameter measurements of the lesions at baseline and follow-up were all above 95%, indicating excellent intra- and inter-examiner reliability (Table 2). The average time required to set the initial outline of the lesion for the preview outline was 39 ± 2 s for observer I and 40 ± 2 s for observer II. The subsequent adjustments to define the final outline took an additional mean of 70 ± 5 s for observer I and 60 ± 5 s for observer II, with a statistically significant difference between the two observers (*p* = 0.02). Descriptive statistics on the lesion dimensions and the relative difference between preview and final dimensions are displayed in Table 3.

**Table 2:** Inter-rater agreement assessed using intraclass correlation coefficients (ICC) for preview and final dimensions on the baseline and follow-up CBCT for the lesions volume, surface and diameter

	ICC Baseline, Preview	ICC Baseline, Final	ICC Follow-Up, Preview	ICC Follow-Up, Final
Volume	96%	98%	96%	97%
Surface	98%	99%	97%	97%
Diameter	95%	96%	96%	96%

**Table 3:** Comparison of the lesions dimensions between the preview and final lesion outline at baseline and follow-up

	Baseline						Follow-Up					
	Volume (mm^3^)		Surface area (mm^2^)		Max. diameter (mm)		Volume (mm^3^)		Surface area (mm^2^)		Max. diameter (mm)	
	**Mean** (95% CI)	**Median** (min, max)	**Mean** (95% CI)	**Median** (min, max)	**Mean** (95% CI)	**Median** (min, max)	**Mean** (95% CI)	**Median** (min, max)	**Mean** (95% CI)	**Median** (min, max)	**Mean** (95% CI)	**Median** (min, max)
**Preview outline** (*n*** = 40)**	**3584** (3057, 4112)	**2996** (806, 11606)	**1316** (1179, 1453)	**1215** (469, 3212)	**29** (27, 32)	**28** (15, 57)	**1356** (1055, 1657)	**772** (36, 6043)	**631** (533, 729)	**507** (66, 2207)	**19** (17, 21)	**17** (7, 49)
**Final outline** (*n*** = 40)**	**3764** (3216, 4311)	**3250** (858, 12127)	**1392** (1246, 1537)	**1266** (494, 3498)	**30** (28, 32)	**28** (15, 58)	**1413** (1099, 1727)	**793** (39, 6307)	**665** (561, 769)	**525** (69, 2276)	**20** (18, 22)	**18** (7, 51)
**Difference (%)** **preview – final**	5.0%	8.5%	5.8%	4.2%	2.3%	0.0%	4.2%	2.7%	5.3%	3.6%	2.6%	2.9%

### Confounding factors on dimensional changes over time

**Fig. 2:**
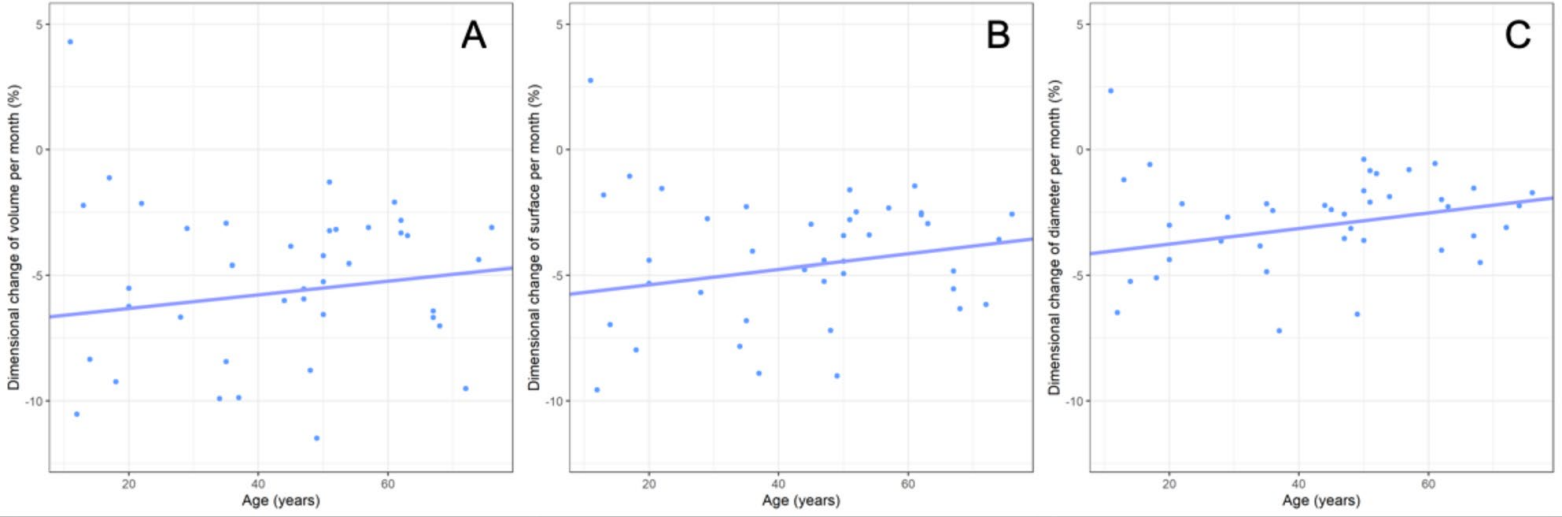
Association between patient age and the lesions` monthly volume (**A**), surface (**B**) and diameter (**C**) reduction

**Fig. 3:**
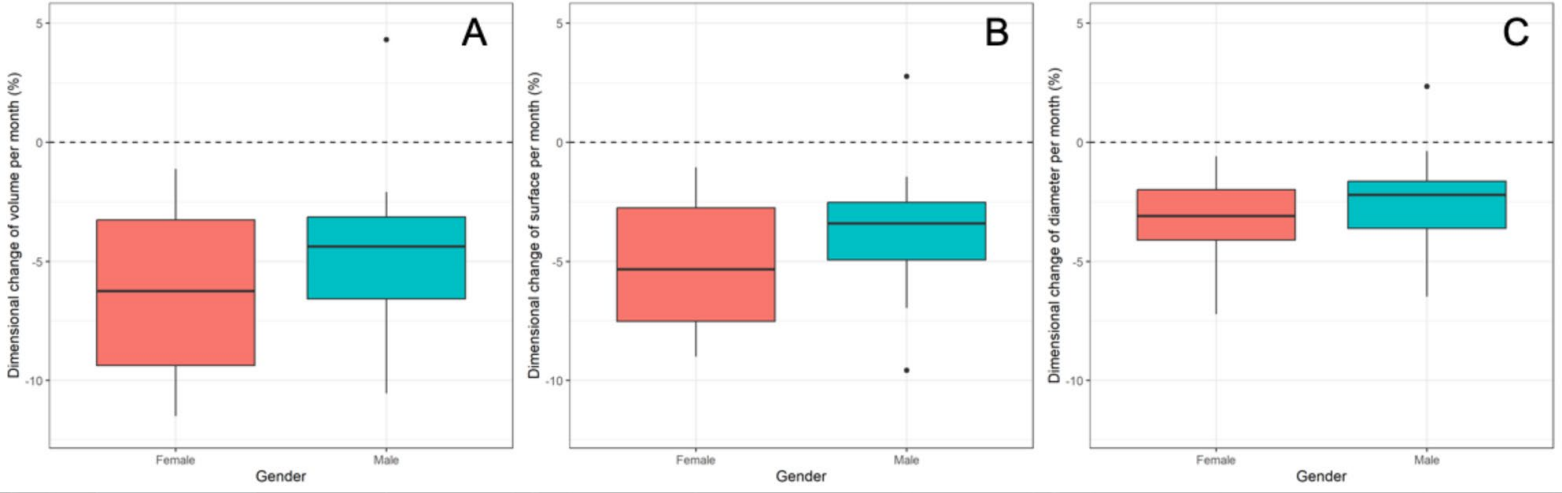
Effects of gender on the lesions` monthly volume (**A**), surface area (**B**) and diameter reduction (**C**)

The overall monthly volumetric, surface area, and maximum diameter reduction rates averaged to −5.2%/−4.3%/−2.8%, with a single case of an OKC showing an increase in dimension of 4.3%/2.8%/2.3%. The dimensional changes over time were not affected by the cyst type, with a mean monthly volumetric, surface area, and maximum diameter change of −6.7%/−5.5%/−3.5% for DC, followed by −4.7%/−4.0%/−2.7% for RC, and − 4.4%/−3.6%/−2.3% for OKC (*p* ≥ 0.14). Higher patient age was significantly associated with less reduction in maximum diameter (*p* = 0.03) but did not significantly affect reduction in volume or surface area reduction (*p* ≥ 0.12, Fig. 2). Female patients showed a greater reduction in cyst volume and surface area (−6.78% and − 5.61%) compared to male patients (−4.96% and − 4.07%, *p* ≤ 0.04), but not in maximum diameter (*p* = 0.11, Fig. 3). Baseline lesion dimensions, lesion location, and the presence of cortical perforations did not significantly affect monthly dimensional changes (*p* ≥ 0.2). Descriptive statistics for lesion dimensions and changes over time, categorized by cyst type, are provided in Table 4, while the results of the regression analysis are shown in Table 5.

**Table 4:** Final outline dimensions at baseline and follow-up and monthly dimensional change rates of the lesions` volume, surface area, and maximum diameter. OKC: odontogenic keratocyst, DC: dentigerous cyst, RC: radicular cyst, BC: botryoid cyst

	All (DC + OKC + RC + BC) *n* = 40						DC (*n* = 14)						OKC (*n* = 15)						RC (*n* = 10)					
**Interval** **Baseline – FollowUp**	Mean (95% CI) 15.7 (11.4, 20.1)			Median (min, max) 12.2 (4.5, 87.1)			Mean (95% CI) 11.9 (7.3, 16.5)			Median (min, max) 9.2 (4.5, 34.0)			Mean (95% CI) 19.5 (8.4, 30.5)			Median (min, max) 12.4 (6.7, 87.1)			Mean (95% CI) 15.7 (11.4, 20.0)			Median (min, max) 12.2 (8.7, 27.8)		
	**Volume ** **(mm3)**		**Surface area ** **(mm2)**		**Max. diameter ** **(mm)**		**Volume ** **(mm3)**		**Surface area ** **(mm2)**		**Max. diameter ** **(mm)**		**Volume ** **(mm3)**		**Surface area ** **(mm2)**		**Max. diameter ** **(mm)**		**Volume ** **(mm3)**		**Surface area ** **(mm2)**		**Max. diameter ** **(mm)**	
	Mean (95% CI)	Median (min, max)	Mean (95% CI)	Median (min, max)	Mean (95% CI)	Median (min, max)	Mean (95% CI)	Median (min, max)	Mean (95% CI)	Median (min, max)	Mean (95% CI)	Median (min, max)	Mean (95% CI)	Median (min, max)	Mean (95% CI)	Median (min, max)	Mean (95% CI)	Median (min, max)	Mean (95% CI)	Median (min, max)	Mean (95% CI)	Median (min, max)	Mean (95% CI)	Median (min, max)
**Baseline**	3764 (3216, 4311)	3250 (858, 12127)	1392 (1246, 1537)	1266 (494, 3498)	30 (28, 32)	28 (15, 58)	3624 (2765, 4484)	3250 (1108, 11237)	1359 (1124, 1594)	1323 (579, 3249)	30 (26, 34)	29 (16, 58)	4481 (3373, 5589)	3922 (1058, 12127)	1576 (1298, 1854)	1452 (631, 3498)	33 (29, 37)	30 (19, 57)	2771 (1998, 3543)	2355 (858, 6218)	1115 (883, 1346)	1142 (494, 2008)	26 (22, 30)	25 (15, 40)
**Follow-Up**	1413 (1099, 1727)	793 (39, 6307)	665 (561, 769)	525 (69, 2276)	20 (18, 22)	18 (7, 51)	1319 (811, 1827)	1034 (96, 6208)	645 (461, 829)	544 (120, 2276)	20 (16, 23)	18 (8, 51)	1753 (1155, 2351)	834 (60, 6307)	783 (595, 971)	575 (113, 1937)	22 (19, 24)	22 (10, 38)	776 (416, 1137)	530 (39, 2844)	439 (317, 561)	344 (69, 999)	16 (13, 19)	14 (7, 32)
**Reduction per month (%)**	− 5.2 (− 4.2, − 6.2)	− 4.9 (4.3, − 11.5)	− 4.3 (− 3.5, − 5.1)	− 4.2 (2.8, − 9.6)	− 2.8 (− 2.2, − 3.4)	− 2.4 (2.3, − 7.2)	− 6.7 (− 5.0, − 8.5)	− 6.6 (− 2.1, − 11.5)	− 5.5 (− 4.0, − 7.1)	− 4.9 (− 1.4, − 9.6)	− 3.5 (− 2.1, − 4.8)	− 2.5 (− 0.4, − 7.2)	− 4.4 (− 2.4, − 6.4)	− 3.4 (4.3, − 9.9)	− 3.6 (− 2.1, − 5.0)	− 3.0 (2.8, − 7.8)	− 2.3 (− 1.4, − 3.3)	− 2.7 (2.3, − 4.4)	− 4.7 (− 3.5, − 6.0)	− 5.1 (− 2.1, − 7.0)	− 4.0 (− 2.8, − 5.1)	− 4.2 (− 1.6, − 6.3)	− 2.7 (− 1.9, − 3.4)	− 2.3 (− 1.0, − 4.5)

**Table 5:** Association between confounding variables on the lesions` dimensional change rates. OKC: odontogenic keratocyst, DC: dentigerous cyst, RC: radicular cyst, BC: botryoid cyst

	Variable	*p*-value	Subgroup	Mean Effect in Percentage Points (95% CI)
Volume (mm^3^)	Cyst Type (BC excluded)	0.14	DC vs. OKC DC vs. RC OKC vs. RC	1.54 (−0.47, 3.55) 2.00 (−0.16, 4.18) 0.47 (−1.73, 2.66)
	Baseline Dimension	0.20	per additional mm3	−0.0003 (−0.0006, 0.0001)
	Patient Age	0.25	per additional year	−0.027 (−0.075, 0.020)
	Gender	0.03*	Female vs. Male	1.91 (0.17, 3.64)
	Location	0.57	Posterior vs. Anterior	0.63 (−1.61, 2.87)
	Cortical perforation	0.79	No vs. Yes	0.27 (−1.79, 2.33)
Surface (mm^2^)	Cyst Type (BC excluded)	0.16	DC vs. OKC DC vs. RC OKC vs. RC	1.36 (−0.33, 3.05) 1.56 (−0.26, 3.38) 0.20 (−1.65, 2.04)
	Baseline Dimension	0.24	per additional mm^2^	−0.0006 (−0.0017, 0.0004)
	Patient Age	0.12	per additional year	−0.031 (−0.070, 0.009)
	Gender	0.04*	Female vs. Male	1.54 (0.09, 3.00)
	Location	0.44	Posterior vs. Anterior	0.73 (−1.14, 2.59)
	Cortical perforation	0.79	No vs. Yes	0.22 (−1.50, 1.95)
Diameter (mm)	Cyst Type (BC excluded)	0.43	DC vs. OKC DC vs. RC OKC vs. RC	0.70 (−0.59, 2.00) 0.78 (−0.61, 2.18) 0.08 (−1.33, 1.49)
	Baseline Dimension	0.83	per additional mm	−0.006 (−0.059, 0.047)
	Patient Age	0.03*	per additional year	−0.030 (−0.059, −0.003)
	Gender	0.11	Female vs. Male	0.90 (−0.21, 2.01)
	Location	0.52	Posterior vs. Anterior	0.45 (−0.94, 1.84)
	Cortical perforation	0.84	No vs. Yes	0.13 (−1.16, 1.41)

## Discussion

The present study aimed to assess the dimensional changes of mandibular jaw cysts over time following decompression surgery and found significant volumetric, surface area and maximum diameter reductions between baseline and follow-up CBCT, with greater reductions in younger, and female patients. The cyst type, the baseline dimensions, the location, and the presence of a cortical bone perforation did not influence the dimensional changes.

The overall monthly volumetric reduction was accounting for − 5.2%, with observed mean reductions of − 6.7% for DC, − 4.7% for RC, and − 4.4% for OKC; however, these differences were not statistically significant. These values are slightly lower than the monthly reduction rates of −6.5% to −8.9% reported in other studies, which were derived by dividing the mean dimensional changes by the decompression interval [9, 10, 24, 27–30]. Notably, no statistically significant differences based on the type of pathology were observed [29, 30]. This contrasts with a smaller number of studies that observed significantly faster reduction in DC than RC [19], in RC than OKC [20], in RC than DC or OKC [15], or in DC than RC or OKC [30]. It is important to note, however, that most studies on dimensional changes following decompression surgery are based on relatively small sample sizes, and/or 2D radiograph analysis, which may contribute to the variability in outcomes. In the present study, decompression surgery was not successful in a single case of an OKC regarding volume reduction. The 12-year old boy had an infection a few days after the decompression surgery and recurrently during the 11 months period of fenestration, which might be a possible explanation of the lack of shrinkage. Another in the literature reported reason for an unsuccessful shrinkage is an obstruction of the lesion’s access; however, this was not observed in the present case [31].

Biological sex was significantly associated with dimensional change rates in the present study, with female patients exhibiting significantly greater reductions in both volume and surface area compared to male patients, although the observed effect was of limited clinical relevance. This contrasts with previous studies, which found higher reduction in males compared to females [32], or reported no association between gender and reduction rates [10, 19, 22, 24, 29, 30]. Additionally, patient age, was significantly correlated with the diameter reduction, with younger patients showing pronounced changes. This observation may be explained by the higher regenerative capacity in younger individuals [33], and aligns with the findings of other authors [22, 24, 25, 29]. Although the effect of age on reduction rates reached statistical significance, its clinical relevance appears minimal, amounting to a decrease of 0.03% points per year of age. Other studies have reported no significant relationship between patient age and dimensional change rates [10, 15, 19, 20, 30].

The baseline dimensions of the lesion were not associated with the dimensional change rate, which is consistent with findings from another study [30]. However, this contrasts with the results of most studies, which report significantly higher reduction in larger cysts compared to smaller ones [9, 14, 17, 19, 20, 22, 25, 32], with the decrease in cyst volume being more pronounced during the first three months of decompression compared to later phases [23].

Interestingly, neither the anteroposterior mandibular location nor the presence of cortical bone perforation affected the dimensional changes over time in the present study. To date, the literature has primarily focused on intermaxillary differences, with one study reporting increased reduction rates in the mandible compared to the maxilla [19]. However, this finding was not confirmed by other studies, which found no association between intermaxillary location and reduction rates [14, 30]. Nonetheless, data on the influence of lesion location remains limited [17].

Notably, decompression surgery did not induce changes in histological diagnosis when comparing the incisional biopsy with the final enucleation, as has been observed in isolated cases [34, 35]. To identify any change in presentation, a histopathological examination might be indicated at each stage of surgery. Although not directly investigated here, it is worth considering that thickening of the cyst walls in response to decompression surgery may aid in detaching the soft tissues of the pathology from the surrounding bone, thereby facilitating complete enucleation during the second-stage surgery [16, 36, 37].

The software features used in the present study provided reliable measurements, as the intra- and interexaminer correlation coefficients were high. This finding is consistent with another study on three-dimensional radiographic evaluation, which reported an intra-class correlation coefficient of 0.95 for cyst volume measurements [9]. The time required for the three-dimensional assessment of the lesions was less than two minutes for both examiners, with a statistically significant time difference of 10 s, which we consider negligible in daily practice. Therefore, this rapid analysis, which provides valuable additional information beyond standard CBCT evaluation, appears to be easily implementable in clinical settings. It can enhance the estimation of the interval needed before second-stage surgery and, consequently, assist clinicians in predicting the overall treatment duration for patients. Moreover, alternative methods, such as interpolarization algorithms or the implementation of artificial intelligence, may further reduce the time required for these virtual patient assessments and treatment planning [12, 38].

Despite the findings, the present study has several limitations. First, although the sample size was calculated to achieve sufficient statistical power, the relatively small number of cases overall—and the fact that one histopathological subgroup (i.e., BC) did not meet the required sample size—represents a notable limitation and restricts the generalizability of the results. We included all cysts available within the defined timeframe that met the strict inclusion criteria; however, larger sample sizes in a prospective study design could provide more robust data and facilitate more nuanced subgroup analyses. Second, conducting the study at a single institution may limit the diversity of the patient population. Consequently, the findings may not be fully generalizable to other healthcare settings or patient populations, as differences in clinical protocols, treatment approaches, or population characteristics could influence outcomes. Third, factors such as the surgeon’s experience, variations in surgical techniques, and patient-specific factors (e.g., comorbidities) were not controlled or analyzed. These variables could potentially confound the results and impact the outcomes. Fourth, the CBCT scans used variable exposure parameters, that may result in inconsistencies in the image resolution, lesion delineation, and volumetric accuracy. It is important to note that CBCT should not be routinely employed during the follow-up of odontogenic cysts that do not involve critical anatomical structures, as clinicians must adhere to the ALADAIP (As Low As Diagnostically Acceptable Indication-oriented Patient-specific) principles to minimize patient exposure to radiation.

Future research, including prospective or multi-center studies with larger sample sizes that investigate confounding lesion characteristics (e.g. defect morphology, presence of infection during treatment), different decompression techniques (e.g. stent vs. tube), and patient-reported outcomes (e.g. postoperative discomfort, willingness to repeat the procedure), may help to better understand the effectiveness of decompression therapy for odontogenic cysts.

## Conclusions

Decompression surgery is an effective first-stage treatment to reduce the dimensions of large mandibular odontogenic cysts. Within a clinically limited range, age and sex influenced dimensional changes, whereas histopathologic cyst type, baseline size, and cortical perforation did not. CBCT-based volumetric analysis using specialized software proved to be a reliable assessment tool.

## Data Availability

The data that support the findings of this study are available from the corresponding author upon reasonable request.

## Abbreviations

BC: Botryoid cyst
CBCT: Cone-beam computed tomography
ICC: Intraclass correlation coefficients
DC: Dentigerous cyst
FOV: Field of view
OKC: Odontogenic keratocyst
RC: Radicular cyst
SD: Standard deviaton
